# Favorable Outcome in Patients with Pheochromocytoma and Paraganglioma Treated with ^177^Lu-DOTATATE

**DOI:** 10.3390/cancers11070909

**Published:** 2019-06-28

**Authors:** Achyut Ram Vyakaranam, Joakim Crona, Olov Norlén, Dan Granberg, Ulrike Garske-Román, Mattias Sandström, Katarzyna Fröss-Baron, Espen Thiis-Evensen, Per Hellman, Anders Sundin

**Affiliations:** 1 Section of Radiology, Molecular Imaging, Department of Surgical Sciences, Uppsala University, Akademiska Sjukhuset, SE-751 85 Uppsala, Sweden; 2 Department of Medical Sciences, Uppsala University, Uppsala University Hospital, SE-751 85 Uppsala, Sweden; 3 Section of Endocrine Surgery, Department of Surgical Sciences, Uppsala University, Uppsala University Hospital, SE-751 85 Uppsala, Sweden; 4 Department of Nuclear Medicine, Sahlgrenska University Hospital, SE-413 45 Gothenburg, Sweden; 5 Department of Gastroenterology, Oslo University Hospital, Rikshospitalet, 0372 Oslo, Norway

**Keywords:** pheochromocytoma, paraganglioma, ^177^Lu-DOTATATE, peptide receptor radiotherapy, PRRT, neuroendocrine tumor, NET, PCC, PGL

## Abstract

Peptide receptor radiotherapy (PRRT) with ^177^Lu-DOTATATE has emerged as a promising therapy for neuroendocrine tumors (NETs). This retrospective cohort study aimed to assess the outcome of PRRT for 22 patients with histopathologically confirmed pheochromocytoma (PCC) and paraganglioma (PGL), of which two were localized and 20 metastatic. Radiological response utilized response evaluation criteria in solid tumors 1.1 and toxicity was graded according to common terminology criteria for adverse events version 4. Median 4 (range 3–11) 7.4 GBq cycles of ^177^Lu-DOTATATE were administered as first-line therapy (*n* = 13) or because of progressive disease (*n* = 9). Partial response (PR) was achieved in two and stable disease (SD) in 20 patients. The median overall survival (OS) was 49.6 (range 8.2–139) months and median progression-free survival (PFS) was 21.6 (range 6.7–138) months. Scintigraphic response >50% was achieved in 9/19 (47%) patients. Biochemical response (>50% decrease) of chromogranin A was found in 6/15 (40%) patients and of catecholamines in 3/12 (25%) patients. Subgroup analysis showed Ki-67 <15% associated with longer OS (*p* = 0.013) and PFS (*p* = 0.005). PRRT as first-line therapy was associated with increased OS (*p* = 0.041). No hematological or kidney toxicity grade 3–4 was registered. ^177^Lu-DOTATATE therapy was associated with favorable outcome and low toxicity. High Ki-67 (≥15%) and PRRT received because of progression on previous therapy could constitute negative predictive factors for OS.

## 1. Introduction

Pheochromocytomas (PCCs) and paragangliomas (PGLs) are rare tumors arising from enterochromaffin cells in adrenal medulla and autonomous ganglia [1]. Up to 70% of PCC and PGL have either germ line or somatic mutations in established disease-driver genes [2,3]. The five-year overall survival (OS) rate of patients with metastatic tumors ranges from 40 to 77% [4]. Surgery is the preferred therapy and may cure or allow a long-term remission in patients with resectable disease [5]. The oncologic treatment arsenal consists of local and systemic radiotherapy as well as systemic chemotherapy; the most commonly used systemic treatment is iodine-131-labeled meta-iodo-benzyl-guanidine (^131^I-MIBG) followed by chemotherapy with cyclophosphamide, vincristine, and dacarbazine [6,7,8].

In neuroendocrine tumors (NETs), peptide receptor radiotherapy (PRRT) with lutetium-177 (^177^Lu)- and yttrium-90 (^90^Y)-labeled somatostatin analogs, such as tyrosine octreotide (TOC) and octreotate (TATE), has become frequently used. Predominantly, ^177^Lu-DOTATATE has been utilized, but also ^90^Y-DOTATOC, with favorable results in NETs [9,10,11]. Furthermore, encouraging results of PRRT have been shown in small cohorts of PCC/PGL patients treated with ^90^Y- DOTATOC [9,12] and with ^177^Lu-DOTATATE [13,14,15,16]. In one study, 20 patients received ^177^Lu-DOTATATE, of whom nine also underwent concomitant chemotherapy with fluorouracil as a radiosensitizer, which in 14 evaluable patients (RECIST 1.1) resulted in disease control ((partial response (PR) + stable disease (SD)) in 12/14 (86%) patients with 39 months median OS [14]. In a second study, ^177^Lu-DOTATATE was combined with capecitabine in 25 patients and in 21 evaluable patients achieving disease control, that is, 21/25 (84%) patients, with median 32 months PFS [15]. A third larger ^177^Lu-DOTATATE study included 12 PCC/PGL patients, of whom eight were evaluable (RECIST 1.1) showing disease control (PR + SD) in 6/8 (75%) with 52 months estimated mean OS [16]. ^90^Y-DOTATOC was administered to 28 PCC/PGL patients with disease control (PR + SD) in 71% [12] and in 11 PCC and 28 PGL patients, who were included in a larger PRRT study, radiological response was achieved in 4/11 (36%) of PCC and 3/28 (11%) of PGL patients with mean 32 and 82 months OS, respectively [9].

In our center, we have performed PRRT with ^177^Lu-DOTATATE since 2005, initially applying the treatment protocol developed by the Rotterdam group [10], administering 4 cycles of 7.4 GBq ^177^Lu-DOTATATE (maximum activity 29.6 GBq). By developing a method for normal organ dosimetry, based on uptake measurements on single photon emission computed tomography/computed tomography (SPECT/CT) performed during PRRT, we were able to more accurately estimate the absorbed radiation doses to the kidneys and bone marrow [17,18]. Since 2007, we have therefore instead applied a dosimetry-guided protocol by which PRRT may be individualized for each patient in order to administer as many cycles as possible until reaching 23 Gy absorbed dose to the kidneys or 2 Gy to bone marrow [17,18].

So far, we have treated approximately 800 patients with various NET types. In a prospective study including 200 of these patients with mixed NET types who underwent dosimetry-guided PRRT, because of disease progression or as first-line therapy, tumor response was achieved in 24% and disease control (complete response (CR) + PR + SD) in 96% with 27 months median progression-free survival in all patients, 33 months in those in whom the absorbed dose to the kidneys reached 23 Gy and 15 months in those in whom it did not [19]. In the present study, we retrospectively describe the outcome from treatment with ^177^Lu-DOTATATE in a relatively large group of patients with PCC/PGL.

## 2. Results

Twenty-two patients with PCC and PGL undergoing ^177^Lu-DOTATATE between 2005 and 2018 were identified and included in the study.

### 2.1. Baseline Patient Characteristics

Baseline patient characteristics are shown in Table 1. There were 22 patients (13 men / 9 women) median age 60 years (range 24–80) at the start of therapy, nine had pheochromocytoma and 13 harbored paraganglioma. Ki-67 index was available for 18 patients and the median result was 11% (range 1–30). Except for PCCs (*n* = 9), the primary tumor localization for the PGLs was retroperitoneum (*n* = 7), neck (*n* = 2), and one PGL each in the liver, kidney, urinary bladder, and cauda equina. All tumors except the two PGLs in the neck were metastatic, predominantly to regional retroperitoneal lymph nodes and with distant metastases to bone and liver.

Genetic syndromes were identified based on information from diagnostic DNA sequencing and interpretation available in the medical records. A diagnosis of neurofibromatosis type 1 was also considered from clinical criteria. Nine patients had a genetic syndrome, *SDHB* or *SDHD*-related PGL in seven, neurofibromatosis type 1 in two, four patients were classified as sporadic cases, and genetic testing had not been performed in nine patients. At baseline, chromogranin A was elevated in 15 patients, normal in four, and missing in three patients. Plasma/Urinary (P/U)-catecholamines and their metabolites were normal in 10 patients and elevated in 12. In these 12 patients, normetanephrine was elevated in all, metanephrines in two patients, and methoxytyramine in four. Ten patients experienced catecholamine-related symptoms, which for two patients worsened during PRRT. The patients underwent PRRT because of tumor progression on previous therapies (*n* = 9) or as first-line treatment (*n* = 13). First-line therapy, in eight patients, was administered because of symptomatic disease and for five patients the indication for treatment was not documented in the patient records. The primary tumor was unresectable in three patients because of proximity to vital structures and in three patients due to tumor size (Table 1). Seven patients were previously included in a prospective dosimetry-tailored PRRT study of 200 patients [19]. Eight patients were alive, 11 were deceased, and three patients were lost to follow-up.

### 2.2. Treatment and Toxicity

The PRRT data and registered toxicity are shown in Table 2. Dosimetry-tailored PRRT was performed in 19/22 patients and the first 3/22 patients received PRRT according to a standard four-cycle protocol. The patients received between 3 and 8 cycles of ^177^Lu-DOTATATE during their first treatment. Upon progression, five patients received salvage therapy with another 2 to 4 cycles. Including salvage therapy, between 3 and 11 cycles were administered. Five patients received 3 cycles, seven patients received 4 cycles, five patients 5 cycles, two patients 2, and one patient each received 7, 10, and 11 cycles, respectively. The median amount of ^177^Lu-DOTATATE activity per cycle was 7.4 GBq and the median total administered activity (including salvage therapy) was 29.6 (range 22.2–81.4) GBq. The reasons to stop PRRT were 23 Gy dose to the kidneys reached (*n* = 17), use of a four-cycle PRRT protocol (*n* = 3), and progressive disease (*n* = 2). Hematological side effects occurred in 16/22 (73%) patients, all classified as grades 1 (*n* = 10) or 2 (*n* = 6) (Table 3). No kidney toxicity was observed.

### 2.3. Therapy Outcome and Response Assessment

**Figure 1:**
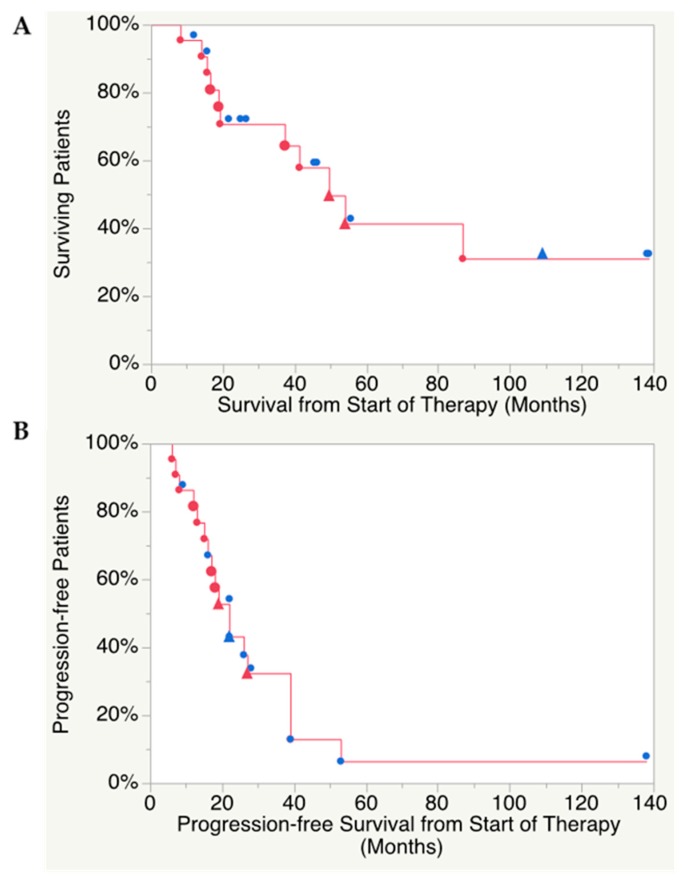
(**A**) Kaplan–Meier analysis of overall survival, median 49.6 (range 8.2–139) months and (**B**) progression-free survival, median 21.6 (range 6.7–138) months. Blue symbols, patient alive; red symbols, patient deceased. Triangles, patient received salvage therapy.

Median follow-up time was 32 (range 8–139) months. Median overall survival (OS) was 49.6 (range 8.2–139) months (Figure 1A) and median PFS was 21.6 (range 6.7–138) months (Figure 1B). Response rates are summarized in Table 3. The median best response on CT according to RECIST 1.1 (%) across the cohort was −10% (range 0 to −65%). The time to best response was median 14.4 months (range 4.7 to 128 months). Best response was partial response (PR) in two patients and the remaining 20 patients reached stable disease (SD), whereas no complete response (CR) was found.

In the 9 patients who were progressive at PRRT start, 1 achieved PR and 8 SD with response according to RECIST1.1 median −14% (range −56 to 0%), as compared to 13 patients who received PRRT as first-line therapy of whom 12 achieved SD and 1 PR with RECIST1.1 response median −16% (range −50 to 0%).

Response on ^177^Lu-DOTATATE-SPECT/CT and whole-body scans was seen in 14/19 (74%) patients and the visually rated decrease in tumor accumulation, as compared to scintigraphy during the first cycle, was median 50% (range 20–65%), resulting in a response rate PR in nine (47%), SD in eight (42%) and progressive disease (PD) in two (11%) patients.

The biochemical response data are shown in Table 3. P/U-catecholamines were available for all patients and were normal in 10/22 (45%). Catecholamine response with >50% reduction was found in 3/12 evaluable patients (25%), >25–50% decrease in 6/12 (50%), >0–25% reduction in 2/12 (17%), and biochemical progression in 1/12 (8%). The biochemical response was consistent with the morphological response rate on computed tomography or magnetic resonance imaging (CT/MRI) (RECIST 1.1), except in one radiologically stable patient who showed biochemical progression and in one patient with partial remission in whom the P/U-catecholamines showed a mere 29% decrease.

Chromogranin A was normal in 5/20 (25%) patients and not available in 2 patients. Biochemical response with >50% reduction of chromogranin A was achieved in 6/15 evaluable patients (40%), >25–50% decrease in 1/15 (7%), >0–25% reduction in 6/15 (40%), and biochemical progression was found in 2 patients (13%). The biochemical response was consistent with the morphological response rate on CT/MRI (RECIST 1.1), except in two radiologically stable patients who showed biochemical progression.

A conflicting biochemical result was found in only one patient with a clear increase of chromogranin A, whereas the catecholamines decreased somewhat in this radiologically stable patient. Of 10 patients with catecholamine-related symptoms during PRRT, these symptoms worsened in 2 patients, improved in 4, and were stable in 4.

The results of two patients with non-metastatic HNPGLs (Nos. 20 and 22, Table 1) were analyzed separately. They did not undergo primary tumor resection because of their tumor’s size and proximity to vital organs/tissue but, previous to PRRT, they had received external radiation radiotherapy. Patient 20 had an SDHD mutation, whereas the other patient was not tested for a genetic profile. Both patients received PRRT as first-line therapy with 4 and 5 cycles, respectively, and achieved −17% and −15% tumor decrease according to RECIST 1.1, resulting in stable disease and 15.6 and 8.6 months PFS and 15.6 and 11.9 months OS, respectively.

### 2.4. Predictors of Outcome

**Figure 2:**
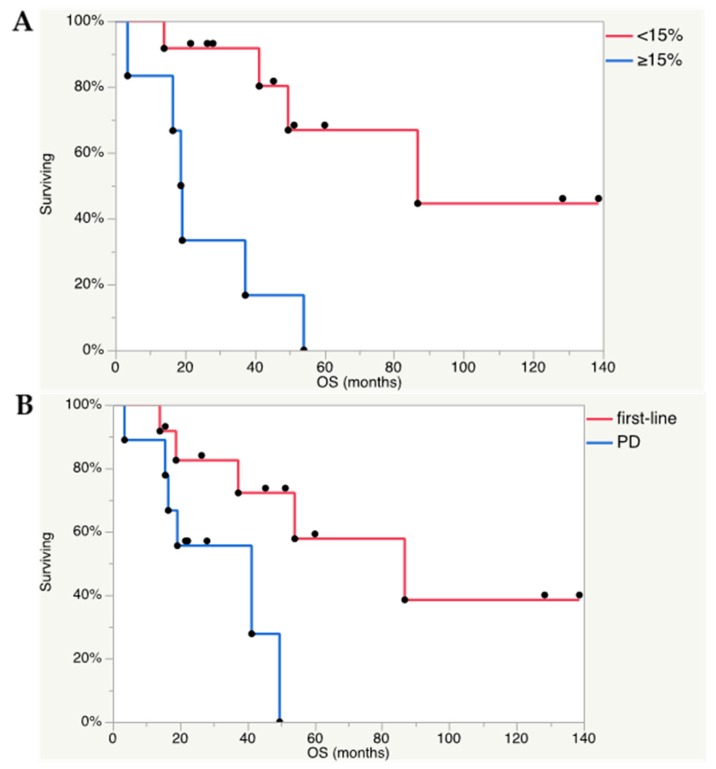
(**A**) Ki-67 index <15% in the Kaplan–Meier analysis was found as a positive predictive factor for OS (Log rank test *p* = 0.013). Out of 18 analyzed patients, 6 had Ki-67 index ≥15% and 12 <15%. Seven patients received PRRT as first-line therapy and 11 because of progressive disease. (**B**) In addition, PRRT administered as first-line treatment, and not because of progressive disease (PD), was found as a positive predictive factor for OS (Log rank test *p* = 0.41). All 22 patients were analyzed and PRRT was administered as first-line therapy in 13 patients and because of progressive disease in 9.

An arbitrary threshold of Ki-67 index 15% was selected as a cut-off. Ki-67 <15% compared to Ki-67 ≥15% (*p* = 0.013) and PRRT received as first-line therapy compared to PPRT received because of tumor progression (*p* = 0.041) were associated with longer OS (Figure 2A,B). Tumor type (PCC or PGL), visual response on scintigraphy (≥50%), biochemical response, number of cycles, administered activity, and previous therapies (surgery, radiotherapy, chemotherapy, ^131^I-MIBG) were factors unrelated to OS. The genetic profile, available for only 15/22 patients, did not allow for analysis as possible predictors of survival.

Ki-67 <15% (*p* = 0.005) was associated with longer PFS, whereas PRRT received as first-line therapy, tumor type, visual response on scintigraphy (≥50%), biochemical response, number of cycles, administered activity, and previous therapies (surgery, radiotherapy, chemotherapy, ^131^I-MIBG) were all factors unrelated to PFS.

## 3. Discussion

We studied the outcome after ^177^Lu-DOTATATE administration in 22 patients with PCC and PGL. Consistent with the results in several reports on PRRT with ^177^Lu-DOTATATE in gastro-entero-pancreatic NETs and lung NETs, the outcome in our patients with PCC and PGL was favorable with disease stabilization in 20 patients and PR in 2 subjects. We observed a median OS of 49.6 (range 8.2–139) months and a median PFS of 21.6 (range 6.7–138) months. Corresponding to two recently published ^177^Lu-DOTATATE studies in small groups of PCC/PGL patients, we had similar duration of follow-up, median 32 (8–139) months, in comparison to the publication by Kong et al. [14] reporting 28 (range 5–74) months follow-up for 14 patients and Yadav et al. [15] who followed 25 patients median 30 (15–96) months after PRRT combined with 1250 mg/m^2^ capecitabine (days 0 to 14 of each cycle). Our follow-up, however, allowed for the calculation of OS, whereas median OS was not reached in the other studies [14,15].

The median PFS 39 months, in the study by Kong et al. and 32 months PFS reported by Yadav et al. were both, however, longer than in the present study (21.6 months). Disease control (PR + SD) according to RECIST 1.1. was achieved in all of our patients as compared to 21/25 (84%) patients and 12/14 (86%) in the two previous reports [14,15], and notably, 11 of our patients were still alive at the time of evaluation. Four of our patients were progression-free between 9 and 138 months. Another ^177^Lu-DOTATATE study, including 12 PGL patients during a median 13 (range 4–30) months follow-up, achieved SD in five and PD in four of nine evaluable patients [13]. In a larger ^177^Lu-DOTATATE therapy study, on mainly high grade (G3) gastroenteropancreatic and lung NETs, 12 patients with PCC/PGL were included of whom eight were evaluable [16]. PRRT resulted in four PR, two SD, and two PD, but in this study the survival data were instead reported as estimated mean OS 51.8 (95% CI 39.8–63.8) months and PFS 31.4 (95% CI 20.3–42.4) months, outcomes which however are difficult to compare to the present results.

A problem potential with utilizing ^90^Y-DOTATOC is the toxicity, as in the study by Imhof et al. [9], who reported transient grade 3–4 myelosuppression in 142/1109 (13%) patients, but in particular kidney toxicity, with severe permanent renal impairment in 102/1109 (9%) patients. Interestingly, no hematological and kidney toxicity greater than grade 1 was experienced by Forrer et al. in 28 patients with surgical incurable PCC/PGL who underwent PRRT with ^90^Y-DOTATOC [12]. With ^177^Lu-DOTATATE, we experienced no kidney toxicity and only grade 1 (*n* = 10) and 2 (*n* = 6) hematological toxicity. Similarly, Yadav et al., who combined ^177^Lu-DOTATATE with capecitabine, reported 3/25 (12%) patients who developed grade 1 lymphopenia, but no other hematological toxicity, and no kidney toxicity [15]. Additionally, Kong et al., who in half of their patients combined ^177^Lu-DOTATATE with fluorouracil, reported mainly grade 2 lymphopenia. However, they also experienced 4/20 (20%) patients with grade 3 lymphopenia. Renal impairment in two of their patients was considered attributed to underlying disease processes [14]. In the larger study by Demirci et al., in 186 patients with mixed NET types, toxicity data for the subgroup of 12 patients with PCC/PGL are not reported. Grade 1 or 2 hematological toxicity was however found in 148/186 (80%) of patients but also 2 (1%) with grade 3 toxicity [16]. No severe kidney toxicity was noted.

The fact that the first three patients were treated according to the original Rotterdam protocol, with four PRRT cycles, and that the remaining patients were instead subjected to a change in protocol where the number of cycles were based on dosimetry, leads to a large variation in the number of administered PRRT cycles and the present results must be interpreted accordingly.

The biochemical responses, regarding chromogranin A and catecholamines, were consistent in all evaluable patients except one in whom a clear increase of chromogranin A was found, whereas the catecholamines decreased somewhat in this radiologically stable patient. Chromogranin A response differed slightly from that of the morphological response rate according to RECIST 1.1. (CT/MRI). Thus, biochemistry indicated PD in two patients with SD on CT/MRI but, in the remaining patients, the biochemical and morphological responses were in agreement. Similarly, the catecholamine response was consistent with the morphological response rate, except in one radiologically stable patient who showed biochemical progression and in one patient with partial remission in whom a mere 29% catecholamine decrease was noted.

Better chromogranin A response was reported by Yadav et al. with >25% reduction in 11/24 (46%) patients of whom >50% reduction was found in 7/24 (29%) [15]. This was also the case in the study by Kong et al. who, out of 14 evaluable patients, found 10/14 (71%) with >25% reduction of chromogranin A, of whom 8/14 (57%) had >50% biochemical response [14]. With ^90^Y-DOTATOC, biochemical responses were reported in 2/11 (18%) and 4/28 (14%) PCC and PGL patients, respectively [9]. We found less biochemical response regarding catecholamines than for chromogranin A. A similar degree of catecholamine response was found by Kong et al., who for 7 patients reported >50% decrease of plasma metanephrine in 1 patient, 25% to 50% reduction in 4 patients, and biochemical increase in 2 patients, and with fairly similar biochemical response figures regarding plasma normetanephrine [14].

Ten of our patients had catecholamine-related symptoms, which in two were aggravated during PRRT, including the development of hypertensive crisis. These symptoms and signs were, however, present already at baseline and it is difficult to confidently determine whether the worsening of symptoms was a consequence of PRRT, or represented fluctuations of catecholamine levels due to the disease itself.

The response to ^177^Lu-DOTATATE therapy was also assessed during each PRRT cycle by scintigraphy, including both whole-body scans and SPECT/CT, whereby a visual rating of the tumor uptake is made for each cycle in relation to the first. In this evaluation, we found response (≥50%) in 9/19 (47%) of our patients who underwent dosimetry and for whom scintigraphic examinations were available. Another means for therapy monitoring was applied by Kong et al., who utilized ^68^Ga-DOTATATE-PET/CT and reported a similar fraction of responding patients (8/17, 47%) [14].

**Figure 3:**
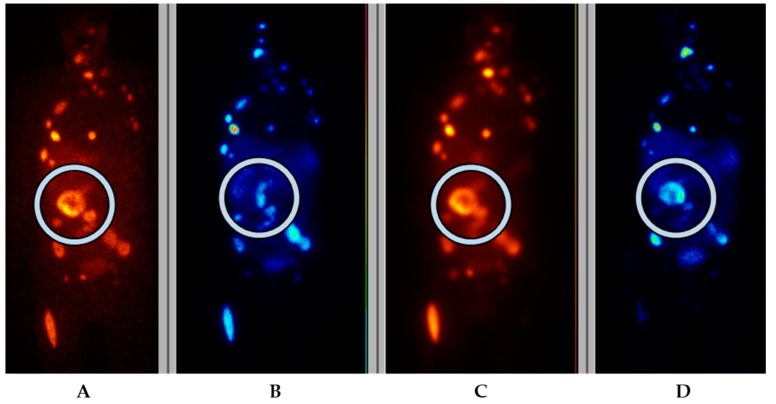
Frontal planar scintigraphy images in patient No. 4, in whom the primary PCC had higher uptake on ^123^I-MIBG scintigraphy (**A**) than on somatostatin receptor scintigraphy (OctreoScan™) (**B**). After one cycle of ^131^I-MIBG (**C**) scintigraphy during therapy) an upregulation of somatostatin receptors was noted and further therapy was given with ^177^Lu-DOTA-octreotate (**D**) scintigraphy during therapy).

^131^I-MIBG therapy constitutes the mainstay in radionuclide therapy of PCC/PGL, and which five of our patients had previously undergone. With ^177^Lu/^90^Y-DOTATATE/TOC therapy, the tumors need to show sufficient somatostatin receptor expression on imaging, usually Krenning grade 3 or 4 (higher than that of the liver) or at lease grade 2 (similar to that of the liver) in order to be eligible for PRRT. Correspondingly, the tumors on ^123^I-MIBG scintigraphy need to show sufficient uptake for the patient to qualify for ^131^I-MIBG therapy. A problem with ^123^I-MIBG in PCC/PGL patients is that the uptake in general is high, resulting in excellent sensitivity on ^123^I-MIBG scintigraphy for diagnosing disease on a patient basis. However, ^123^I-MIBG scintigraphy has shown reduced sensitivity in extra-adrenal, multiple, or hereditary PGLs (52–75%) associated with von Hippel Lindau syndrome as well as *SDHB*-related PGLs and patients with metastatic disease in whom ^123^I-MIBG scintigraphy may underestimate the extent of disease. The sensitivity of ^123^I-MIBG scintigraphy is particularly low in head and neck PGLs (HNPGL) (18–50%) [20]. Consequently, the low tumor accumulation on ^123^I-MIBG scintigraphy disqualifies many of these patients for ^131^I-MIBG therapy. Our patients underwent both ^123^I-MIBG scintigraphy and somatostatin receptor scintigraphy, and first-line therapy was chosen based on these results. In the case of inhomogeneous expression, the tracer with the most favorable distribution was applied for subsequent therapy (Figure 3). In addition, patient No. 3 had a mosaic expression for the two tracers, which resulted in treatment with both, consecutively. One lesion without any uptake was treated with additional external beam radiation.

The concept of Ki-67 proliferation index as a predictive marker of response to treatment in patients with NET in general, and in NET patients treated with ^177^Lu-DOTATATE in particular, is well established and part of clinical routine. In PCC and PGL, Ki-67 index is, however, not validated as a predictive marker. Interestingly, we found such a correlation and Ki-67 ≥15% was shown to be a predictor for worse OS and PFS. Another negative predictor for OS, but not for PFS, was found to be PRRT undertaken because of progression on previous therapy, and not started as a first-line treatment. We were however unable to show any predictive value for tumor type (PCC or PGL), visual response on scintigraphy, or previous therapies (surgery, radiotherapy, chemotherapy, ^131^I-MIBG), which were all factors unrelated to OS and PFS. Most probably, because of the small number of observations, neither was the patients’ genetical profile or disease stage useful as predictors of survival.

In three of our patients, the PGL was located in the cauda equina, liver, and kidney, respectively. Although PGL in these anatomical positions are extremely uncommon, they were, because of previously published reports on PGLs in these sites, regarded as primary tumors. It can of course not be confidently excluded that these tumors represented metastases from an undiagnosed PGL with unknown primary location.

## 4. Materials and Methods

This was a retrospective cohort study of patients treated at the Department of Nuclear Medicine, Uppsala University Hospital, Uppsala, Sweden. All patients receiving ^117^Lu-DOTATATE therapy between 2005 and 2018 were screened for inclusion using information available through the digital radiological information and picture archive and retrieval systems (RIS-PACS). The study was approved by the local ethics committee, and written informed consent was obtained from all individual patients since 2010 (No. 2009-320). Before 2010, patients were admitted after giving their informed consent on a single-patient basis for compassionate use with individual permission of the Swedish Medical Products Agency.

Clinical, biochemical, and radiological imaging follow-up data were retrieved from the RIS-PACS and from the hospital’s digital patient record system. Survival was analyzed for Swedish patients (*n* = 15), based on entries to the national health registry accessed on 5 April 2019. For international patients, the referring center was contacted for an updated follow-up.

Inclusion criteria for PRRT were patients with pheochromocytoma or paraganglioma confirmed by histopathological examination, and a tumor uptake higher than that of the normal liver (Krenning scores 3 and 4) on somatostatin receptor scintigraphy (SRS), life expectancy > 3 months, white blood cell count (WBC) > 3.0 × 10^9^/L, platelet count > 100 × 10^9^/L, bilirubin < 40 µmol /L, albumin > 25 g/L, ASAT and ALAT less than 5 times upper limit, creatinine < 110 µmol/L or, if higher, GFR (cystatin-C) > 50 mL/min/1.73 m^2^. Exclusion criteria were pregnancy, tumors amenable to surgery or locoregional ablation, and inability to stay isolated for 24 h.

### 4.1. PRRT Treatment Protocol

Chemotherapy and targeted therapy were stopped at least one month before the treatment start. Therapy monitoring utilized intravenously contrast-enhanced computed tomography (CT) or magnetic resonance imaging (MRI) performed according to clinical NET imaging protocols within one month before PRRT, in connection with every second cycle, 3 months after the end of treatment, and thereafter every 3–6 months until tumor progression.

Three patients were treated according to the original Rotterdam PRRT protocol with 4 cycles × 7.4 GBq ^177^Lu-DOTATATE [10] and 19 patients underwent dosimetry-guided PRRT [19], whereby as many cycles as possible were administered with an intended interval of 6 to 8 weeks between cycles up to 23 Gy to the kidneys or 2 Gy to bone marrow, or other reasons to stop therapy occurred.

Dosimetry for solid organs and bone marrow was calculated as previously described in detail [17,18]. For solid organs, dosimetry was based on the small volume method performed on single photon emission tomography with low dose CT performed together with single photon computed emission tomography (SPECT/CT) at 1, 4, and 7 days after therapy. Volumes of interest (4 mL) were drawn in representative regions with homogeneous uptake. The activity concentrations were fitted to a mono-exponential function. For complete bone marrow dosimetry, the blood activity was calculated from integrated blood activity curves derived from blood samples at 0.5, 1, 2.5, 4, 8, and 24 h and complemented with tissue activity measurements on whole-body scans at 1, 4, and 7 days after start of therapy. Complete dosimetry was performed during the first cycle, after delay, in the instance of large changes in tumor volume, and at least at every fourth cycle. For all other cycles, a short dosimetry protocol used SPECT/CT over the abdomen and a whole-body scan at 24 h.

The peptide was a kind gift from Prof. Eric Krenning, ^177^Lu was purchased (IDB, Holland BV, Baarle-Nassau, The Netherlands) and labeling was performed in-house. Kidney protection comprised 2 L amino acid mixture (Vamin 14 gN/L electrolyte-free, Kabi Fresenius) i.v. over 8 h, starting 30 m before treatment. I.v. antiemetics was given 1 h before therapy (8 mg of betamethasone and 8 mg of ondansetron or 250 µg of palonosetron).

### 4.2. Evaluations

White blood cell (WBC) count was checked before each cycle and had to be >3 × 10^9^/L, granulocytes >1.5 × 10^9^/L, and platelets >100 × 10^9^/L. If these criteria were not met within 6 months, PRRT was stopped. In occasional instances, the interval between cycles was prolonged, alternatively the administered activity per cycle was decreased by 30%, rather than delaying the treatment. Plasma chromogranin A as well as plasma/urinary metanephrines or catecholamines were collected in connection with every cycle. In order to monitor side effects, routine blood tests (complete blood count, liver enzymes, electrolytes, and creatinine) were performed every second week on an outpatient basis. CT/MRI was performed within one month before PRRT start, before every second treatment cycle, 3 months after the last treatment, and thereafter at least every 6 months.

### 4.3. Response Assessment

CT/MRI results were assessed according to RECIST 1.1. [21] to determine the best tumor response (%) (BR%) and response category (complete response (CR), partial response (PR), stable disease (SD), and progressive disease (PD)) and the time to BS from start of PRRT. The progression-free survival (PFS) and overall survival (OS) were calculated.

Biochemical tumor response of P-chromogranin A and P/U-catecholamines was registered in four categories, namely, decrease >50%, decrease >25–50%, decrease >0–25%, or increase >50%, as compared to baseline values.

SPECT/CT and whole-body scans and SPECT during each cycle were evaluated by a specialist in nuclear medicine, whereby the overall decrease in tumor uptake (%) on scans performed during each PRRT cycle was assessed in relation to that of the first cycle. PR was defined as ≥50% decrease and PD was registered in the case of pronounced increase in tumor accumulation or appearance of new tumor lesions.

### 4.4. Toxicity

Toxicity was reported using the Common Terminology Criteria for Adverse Events version 4.0, and toxicities grade 1 to 4 were reported from the start of treatment until 3 months after the last treatment.

### 4.5. Statistical Analysis

Univariate correlation to PFS and OS was performed with JMP 13.1.0 (SAS Institute Inc., Cary, NC, USA) using the Kaplan–Meier log rank test. Possible predictive factors for OS and PFS were tested in the Kaplan–Meier analysis by dichotomization. Multivariate analysis was excluded due to the limited sample number.

## 5. Conclusions

In conclusion, PRRT with ^177^Lu-DOTATATE was associated with a favorable outcome and low toxicity. High Ki-67 (≥15%) and PRRT received because of progression on previous therapy were negative predictive factors for OS.

## Figures and Tables

**Table 1:** Baseline patient characteristics.

Pat. No.	Age at PRRT Start	Sex	Tumor Type	Primary Tumor Localization	Ki-67 Index	Genotype	Indication for PRRT	Metastases	Previous Therapy			
									Surgery	RT	^131^I-MIBG	ChT
1	61	F	PGL	Retroperitoneum	5%	*SDHB*	Sympt	Retroper lgll, Liver, Bone	+	+	-	-
2	33	F	PGL	Urinary bladder	10%	*SDHD*	NA	Mediast lgll, Neck, Heart	+	-	-	-
3	24	M	PGL	Retroperitoneum	<2%	Sporadic	Sympt	Retroper lgll, Bone	+	+	+	-
4	67	M	PGL	Aortic bifurcation	15%	*SDHB*	PD	Bone	-	+	+	-
5	53	F	PCC		2%	Sporadic	Sympt	Liver	+	-	-	-
6	25	M	PGL	Retroperitoneum	NA, 4/10HPF	NA	PD	Bone	+	+	-	+
7	56	M	PCC		12%	NA	PD	Retroper lgll, Bone	+	+	-	-
8	71	F	PGL	Liver	25%	Sporadic	NA	Bone	+	-	-	-
9	70	F	PGL	Kidney	20%	NA	PD	Bone	+	+	+	-
10	25	M	PGL	Retroperitoneum	25%	*SDHB*	NA	Liver, Bone, Lung	-	-	-	-
11	56	F	PCC		30%	*NF1*	PD	Liver, Bone	+	-	-	-
12	59	F	PGL	Retroperitoneum	NA	NA	PD	Bone, Mediastinal lgll	+	+	+	-
13	55	M	PCC		22%	NA	Sympt	Bone	-	+	-	-
14	65	F	PGL	Cauda equina	13%	Sporadic	PD	0	+	+	-	-
15	62	M	PCC		<1%	NA	Sympt	Liver, Bone, Lung	+	+	-	-
16	67	M	PGL	Aortic bifurcation	5%	NA	PD	Liver, Bone	+	-	-	-
17	80	M	PCC		<1%	*SDHA*	Sympt	Retroper lgll. Liver, Lung, Bone	+	-	+	-
18	39	F	PCC		3%	*SDHB*	NA	Retroper and mediastinal lgll, Lung	-	+	-	-
19	72	M	PCC		1%	NA	NA	Liver, Bone	+	-	-	-
20	63	M	HNPGL	Bilateral neck	NA	*SDHD*	Sympt	0	-	+	-	-
21	79	M	PCC		NA	*NF1*	PD	Bone	+	+	+	-
22	31	M	HNPGL	Bilateral neck	NA	NA	Sympt	Liver, Bone	-	+	-	-

F, female; M, male; PCC, pheochromocytoma; PGL, paraganglioma; HNPGL, head and neck paraganglioma; NA, not available; Sympt, symptomatic; Retroper lgll, Retroperitoneal lymph node metastases; Mediast lgll, Mediastinal lymph node metastases: RT, Radiotherapy; ChT, Chemotherapy.

**Table 2:** Number of administered ^177^Lu-DOTATATE cycles and hematological toxicity.

Patient No.	No. PRRT Cycles First Treatment	No. PRRT Cycles Salvage Treatment	No. PRRT Cycles in Total	Hematological Toxicity		
				Trbc Toxicity Grade	RBC Toxicity Grade	WBC Toxicity Grade
1	3		3	0	1	1
2	4		4	0	0	0
3	4		4	0	2	0
4	4		4	0	0	0
5	4	3	7	0	0	2
6	4		4	0	0	0
7	6	4	10	0	1	0
8	4		4	0	0	2
9	3		3	0	0	0
10	8	3	11	0	0	2
11	3		3	1	1	0
12	5		5	0	2	0
13	6		6	0	1	0
14	3	2	5	0	2	0
15	4		4	0	0	0
16	5		4	0	0	0
17	5		4	0	1	0
18	4	2	6	0	1	0
19	3		3	1	0	1
20	5		5	0	1	0
21	3		3	0	1	0
22	4		4	0	1	1
Sum	94	14	108	*n* = 2 grade 1	*n* = 9 grade 1 *n* = 3 grade 2	*n* = 3 grade 1 *n* = 3 grade 2

PRRT, peptide receptor radiotherapy; Trbc, thrombocytes; RBC, red blood cell; WBC, white blood cell.

**Table 3:** **** Response rates for peptide receptor radiotherapy (PRRT) with ^177^Lu-DOTATATE in 22 patients with pheochromocytoma or paraganglioma (PCC/PGL).

Patient No.	NM Response ≥50%	Best Response RECIST 1.1 (%)	Best Response RECIST 1.1 (Category)	Time to BR RECIST 1.1 (Months)	OS (Months)	PFS (Months)	Catecholamine Response	CgA Response
1	NA	−17	SD	6.7	86.8	6.7	−48% *	NA
2	NA	0	SD	128.4	138.2	138.2	Normal	Normal
3	NA	0	SD	45.2	139.2	53	−43% *	−61
4	Yes	−13	SD	4.7	8.2	8.2	Normal	−21
5	Yes	−18	SD	15.2	109.4	22.5	−28%	−35
6	No	0	SD	6.5	21.6	21.6	Normal	Normal
7	Yes	−2	SD	15.0	49.6	27	−38% *	−5
8	Yes	0	SD	12.0	37.3	16.7	Normal	−51
9	No	−14	SD	11.3	19.2	5.6	−14%	164
10	No	−29	SD	13.8	54.1	18.8	Normal	−85
11	No	−15	SD	9.8	16.5	11.8	Normal	NA
12	Yes	−65	PR	10.5	15.6	14.6	−81%	−15
13	No	−6	SD	15.6	18.8	18.3	−43%	−52
14	Yes	−18	SD	15.0	41.3	38.9	Normal	Normal
15	Yes	−50	PR	9.5	14	12.7	−29%	−6
16	Yes	−6	SD	15.8	55.6	39.1	315%	138
17	No	−7	SD	20.0	45.4	28	−53%	−56
18	No	0	SD	8.2	26.4	26.4	Normal	−14
19	Yes	0	SD	32.8	46.2	38.8	−13%	−55
20	No	−7	SD	15.5	15.6	15.6	Normal	Normal
21	No	−15	SD	16.4	24.9	22.3	−89% *	−1
22	No	−15	SD	8.6	11.9	8.6	Normal	Normal

NM response, response on scintigraphy during PRRT; NA, not available; PR, partial response; SD, stable disease; PD, progressive disease; BR, best response; OS, overall survival; PFS, progression-free survival; CgA, chromogranin A; Normal, within or not higher than 10% above the upper reference value. Catecholamines were measured in plasma except *, in urine.
